# Daptomycin-Rifampin-Induced Rhabdomyolysis, Acute Renal Failure, and Hepatic Injury: A Case Report and Literature Review

**DOI:** 10.7759/cureus.36834

**Published:** 2023-03-28

**Authors:** Venu M Ganipisetti, Natasha Dudiki, Anand Athavale, Pratyusha Bollimunta, Babu Sriram Maringanti

**Affiliations:** 1 Hospital Medicine, Presbyterian Hospital, Albuquerque, USA; 2 Pulmonary and Critical Care Medicine, Indiana University Health Ball Memorial Hospital, Muncie, USA; 3 Hospital Medicine, University of New Mexico, Albuquerque, USA

**Keywords:** rifampicin, daptomycin rifampin, rifampin, chronic kidney disease (ckd), overweight and obesity, drug-induced liver injury (dili), rhabdomyolysis, drug-induced myopathy, drug induced acute kidney injury, daptomycin side effects

## Abstract

Daptomycin is a canonical antibiotic used very commonly in practice for its bactericidal activity against Gram-positive bacteria, including vancomycin-resistant enterococci (VRE) and methicillin-resistant Staphylococcus aureus (MRSA) bacteremia, bone infections, skin and soft tissue infections, meningitis, urinary tract infections, and endocarditis. Although daptomycin in conventional doses is usually well tolerated, it is paramount to be aware of the possible adverse effects. Daptomycin is reported to cause an elevation in creatine kinase levels, although frank rhabdomyolysis is rare. An even more infrequent occurrence is the simultaneous development of acute kidney injury and drug-induced liver injury with rhabdomyolysis. Daptomycin and rifampin combination are used for synergistic bactericidal action against MRSA. Still, data on the efficacy and safety of the combination is limited due to a lack of extensive studies. Herein, we present a clinical case of septic arthritis of a prosthetic knee, which resulted in bacteremia caused by methicillin-resistant Staphylococcus aureus (MRSA) and subsequently led to infective endocarditis of the aortic valve. The patient was treated with a combination of daptomycin and rifampin, complicated by the development of rhabdomyolysis, acute kidney injury, and drug-induced liver injury. This case highlights the significance of timely recognizing adverse drug effects and identifying risk factors to ensure successful patient outcomes.

## Introduction

Bio-implants are frequently used in the management of degenerative joint disease. Although rare, prosthetic joint infections can significantly affect morbidity and mortality. Bacteria like methicillin-resistant Staphylococcus aureus (MRSA) can form biofilms that help them adhere to the prosthesis surface, evade the immune system and cause persistent infections. 

The Infectious Diseases Society of America (IDSA) has recommended daptomycin as one of the antibiotics for treating MRSA bone and joint infections and native valve infective endocarditis [1]. Rifampin has been used in combination with daptomycin due to its activity against biofilms [2-5]. 

Although this regimen has evidence for use, data on the risks of combination therapy is limited. Herein, we present the case of a patient who developed rhabdomyolysis, acute renal failure, and acute liver injury within 11 days of the institution of this combination therapy; discuss the existing data from the literature and potential risk factors of developing this complication; and highlight the role of further studies.

## Case presentation

An 80-year-old male recently diagnosed with methicillin-resistant Staphylococcus aureus (MRSA) bacteremia, right prosthetic knee septic arthritis, and aortic valve infective endocarditis presented from a rehabilitation center after his follow-up labs in rehab seven days post-discharge showed significantly elevated creatinine kinase (CK) levels along with generalized weakness. His other significant comorbidities include chronic kidney disease stage III (baseline creatinine: 1.1-1.4 mg/dL), coronary artery disease, chronic systolic congestive heart failure (ejection fraction of 35-40%), atrial fibrillation, pacemaker, pituitary insufficiency with adrenal insufficiency, hypothyroidism, chronic hyponatremia (baseline: 126-130 mmol/L). 

Current medications include daptomycin 600 mg intravenous (IV) daily (6 mg/kg/Q24 hour), rifampin 300 mg per oral (PO) twice daily, aspirin, lisinopril, pantoprazole, tamsulosin, and PO hydrocortisone, levothyroxine, and subcutaneous heparin. On exam, his vitals were within normal range, regular rate and rhythm with no audible murmurs on auscultation, clear lung sounds, right knee with intact surgical incision and no drainage or erythema, bilateral lower extremity 2 + pitting pedal edema. He had a restricted range of motion with knee extension and flexion on the right lower extremity. His body mass index (BMI) was 28 Kg/m2, with a body weight of 100 Kg. Admission labs are summarized in Table 1. 

**Table 1:** Admission labs. CO2: Carbondioxide; BUN: Blood urea nitrogen; AST: Aspartate aminotransferase; ALT: Alanine aminotransferase; ALP: Alkaline phosphatase; CK: Creatinine Kinase, hpf: high power field

Lab (Reference range)	Result
Sodium (135-145 mmoL/L)	124
Potassium (3.5-5.1 mmol/L)	3.6
Chloride (98-107 mmol/L)	88
CO2 (19-29 mmol/L)	25
Anion gap (< 18 mmol/L)	16
BUN (7-25 mg/dL)	14
Creatinine (0.7-1.35 mg/dL)	1.83
Calcium (8.6-10.2 mg/dL)	8.3
Albumin (3.3-5.2 gm/dL)	2.3
AST (10-50 U/L)	367
ALT (10-55 U/L)	81
ALP (40-129 U/L)	72
Total Bilirubin (0.0- 1.2 mg/dL)	1.6
CK (39-308 U/L)	7940
Urinalysis	Dipstick positive for blood, microscopic hematuria (9/hpf) without pyuria, proteinuria or casts

During prior hospitalization, he presented with a two-week history of increasing right knee pain and a two-day history of generalized weakness and confusion. Further workup identified sepsis from right prosthetic knee septic arthritis with cultures growing MRSA, leading to MRSA bacteremia and aortic valve endocarditis. The orthopedics team performed surgical exploration, surgical debridement, and polyethylene exchange. Cardio-thoracic surgery team recommended conservative management due to high perioperative morbidity and mortality risk. He was initially treated with vancomycin and transitioned to 600 mg/day daptomycin dosage four days before discharge to rehab. Oral rifampin was added seven days before discharge to rehab for synergistic action to treat the prosthetic joint infection. He was also diuresed to treat volume overload and mild congestive heart failure exacerbation, but diuretics were discontinued on discharge for unknown reasons. The baseline CK level checked was within normal range, and atorvastatin was discontinued due to the risk of drug interaction with daptomycin. A follow-up lab at the rehab seven days after discharge (11 days after starting daptomycin) showed a significantly elevated CK level prompting this admission. 

Both rifampin and daptomycin were discontinued and transitioned to IV vancomycin alone. He was treated with cautious intravenous normal saline (rate of 50 cc/hour) due to concurrent hyponatremia and fluid overload. CK levels and liver function tests (LFTs) normalized within 11 days, and renal failure stabilized to his baseline within a few days. His sodium level improved to 130, and he was discharged back to rehab. Baseline labs at the start of daptomycin and the trend of abnormal labs since the discontinuation of daptomycin-rifampin are summarized in Table 2. 

**Table 2:** Daptomycin and rifampin were discontinued on day 1 of hospitalization. Lab trend from day 1 of daptomycin-rifampin discontinuation to day 11. CK: Creatinine Kinase; AST: Aspartate Aminotransferase; ALT: Alanine Aminotransferase

Lab (reference range)	Daptomycin initiation (baseline labs)	Day 1	Day 2	Day 3	Day 4	Day 5	Day 6	Day 7	Day 8	Day 9	Day 10	Day 11
CK (39-308 U/L)	59	7940	8945	8090	6543	4923	2401	1284	752	547	392	58
AST (10-50 U/L)	34	304	367	404	393	330	229	164	131	113	100	116
ALT (10-55 U/L)	13	65	81	97	118	109	90	78	71	72	65	70
Creatinine (0.7 -1.35 mg/dL)	1.18	1.83	1.77	1.87	1.65	1.66	1.63	1.58	1.56	1.49	1.47	1.42

## Discussion

Daptomycin is a cyclic lipopeptide antibiotic that was food and drug administration (FDA) approved in 2003. It is used to treat Gram-positive infections, including vancomycin-resistant enterococci (VRE) and MRSA bacteremia, skin and soft tissue infections, bone infections, meningitis, urinary tract infections, and endocarditis. This medication has bactericidal activity through various mechanisms, including disruption of cell membrane function, inhibition of DNA and RNA synthesis, and inhibition of protein synthesis. In vitro studies have shown it is very effective and quick against MRSA and methicillin-sensitive Staphylococcus aureus (MSSA), with >99.9% of bacteria dead in 1 hour. Simulated endocardial vegetation models have also demonstrated high bactericidal activity against MSSA and MRSA present in high density within 24 hours. The medication has a longer half-life and can be used with convenient once-a-day dosing. Its clearance is affected by patient characteristics such as sex and body temperature, and it is cleared renally, with 52% of the medication excreted unchanged in the urine [6]. Rifampin has activity against MRSA and has demonstrated synergistic bactericidal action with daptomycin and other antibiotics against MRSA. It is also added to prevent the emergence of resistance [2-5]. Due to the lack of extensive controlled studies, more efficacy and safety data on combining rifampin with daptomycin is essential.

Daptomycin increases CK levels in some patients, although clinically significant rhabdomyolysis is rare. The exact mechanism of myopathy with CK elevation is unknown, although it is hypothesized that it can cause pore-like formations on rhabdomyocytes that are intertwined with cellular membrane lipids. This can induce depolarization and subsequent cellular lysis [7]. Daptomycin causing renal failure and hepatic injury, concurrently with rhabdomyolysis, is an even rarer entity. We performed a literature search in PubMed using the keywords 'Daptomycin,' 'myopathy,' and 'rhabdomyolysis' to identify relevant case reports and also performed citation tracking to identify additional cases. We identified only a few case reports in the literature associated with daptomycin causing acute renal failure, liver injury, and rhabdomyolysis altogether. This is reported only in four patients so far [7-10], which is summarized in Table 3. A few other cases were reported with daptomycin causing rhabdomyolysis and renal failure without liver injury, and a few other cases with daptomycin-induced rhabdomyolysis and liver injury without renal failure. Interestingly, there was also a case report of daptomycin causing renal failure and liver injury without elevation in CK level [11]. Most patients developed symptoms such as weakness, muscle aches, and changes in functional status. The onset of rhabdomyolysis occurred 4-17 days after starting daptomycin when associated with liver injury or renal failure. Almost all patients improved symptoms in a few days with discontinuing daptomycin and supportive treatment, with labs normalizing entirely within a few days to a month. We did not find any instances resulting in this complication with concurrent use of the daptomycin-rifampin combination. 

**Table 3:** Case reports in the literature with Daptomycin-induced acute kidney injury, liver injury, and rhabdomyolysis concurrently. A: Age; S: Sex: N: Number of cases; CK: Creatinine Kinase; CKD: chronic kidney disease; DM: Diabetes mellitus; HTN: Hypertension; Afib: Atrial fibrillation, AKI: Acute kidney injury; CK: Creatine kinase; AST: Aspartate aminotransaminase; ALT: Alanine aminotransaminase; ALP: Alkaline phosphatase; LFT: liver function tests; BMI: body mass index; Cr: Creatinine; f/u; Follow up; IVF: IV fluids; PCKD: polycystic kidney disease; rbc: red blood cells, PVD: peripheral vascular disease; MRSA: methicillin-resistant Staphylococcus aureus; VRE: vancomycin-resistant enterococci.

Authors	A/S/N	Indications, dose	Other significant medications	Comorbidities	Presenting symptoms	CK level	Other significant labs	Treatment/outcome
Nishimura Y [7]	65/M/1	MRSA osteomyelitis. Daptomycin dose: 480 mg/day(6 mg/kg/day)	Lipitor 80 mg/day	Type 2 DM, CKD IIIa (baseline creatinine: 1.0 mg/dL) HTN, dyslipidemia, Afib.	Generalized weakness for 2 days. Bilateral pedal edema present.	Baseline CK: 55 IU/L. CK 9 days after initiation:19460 U/L (39-308)	AKI (Creatinine increased to 1.8), hyperkalemia, AST: 525 (0 to 40) ALT 132 (0-41)	Emergent treatment of hyperkalemia, aggressive IV fluids. Discontinued daptomycin. CK improved to 4100 U/L by day 6. LFTs were also down-trending by day 6. Discharged on linezolid.
King ST el al. [8]	46/F/1	Non-Healing wounds infection, Unclear bacteria. Empiric treatment. Dose: 700 mg/day(6mg/kg/day)	Glipizide,metformin,olmesartan, amlodipine.	Type 2 DM, HTN, possible CKD (Baseline Cr 1.4), obesity with BMI 36/Kgm2	Initially presented with a non-healing wound. Switched to daptomycin on day 5. On the 14th day of initiation, identified with rhabdomyolysis. Had 2 ER visits in between. Initially presented on day 9 with body aches, but labs were normal, and then had a 2nd ER visit and discharge. Forty-eight hours later identified to have abnormal labs.	No baseline CK. CK went upto 16710 U/L.	Cr up to 4.7 mg/dL AST increased to 750, ALT 236, ALP 207. Normal bilirubin.	Treated with IV fluids and discontinuation of Daptomycin. In 5 days - creatinine was down-trending to 2.02; in 1 month, creatinine was down to 1. CK peaked on Day 3 to 20000, down-trending to 7467 on discharge (day 5) AST down-trending. All labs normalized in 1-month f/u. Switched to doxycycline and ciprofloxacin from daptomycin.
Kazory A et al. [9]	53/F/1	Low back pain with discitis failed 8-week IV antibiotic therapy. cultures grew VRE, Toprolus Glabrata, MRSA. Daptomycin dose: 6 mg/kg/day	Voriconazole 250 mg BID	DM, HTN, PVD	Ten days after initiation, muscle weakness and unable to get up.	CK: 21243U/L (unknown baseline)	Urine analysis is positive for myoglobin, rbc. Cr up to 27 mg/L (2.7 mg/dl) from 9 mg/L. LDH increased to 666, AST 375, ALT 219 (increased).	IVF, urine alkalization. Labs normalized -unknown time frame of improvement.
Patel SJ et al. [10]	68/F/1	VRE bacteremia. Dose: 350mg/d (5mg/kg/day)	Tacrolimus, azathioprine, prednisone (40/day), sertraline, folic acid, ursodiol.	PCKD (history of renal transplant), HTN, polymyalgia rheumatica.	On day 4, she developed muscle weakness and was unable to lift her arms,	CK baseline: 17 U/L (25-200); CK went up to 25234;	AST 1155; ALT 332; Cr 1.6 (1.2-1.4), but not clear if daptomycin further worsened this.	Supportive treatment. By day 10, CK decreased to 290, liver enzymes normalized.

Daptomycin myopathy might be dose or frequency related. In phase 1 trials, two out of five patients developed clinically significant myopathy with myalgias and CK elevation when used at 4 mg/kg/q12 hours [12]. This happened less frequently in further studies using a 4 mg/kg/q24 hours dosage. Of 534 patients in a study receiving daptomycin, only 11 had CK elevation, but only two had clinically significant elevation. One of the patients had intramuscular injections and recent surgery with significant elevation on day 9 but no associated symptoms. The other patient had mild to moderate muscle weakness on day 10 but had concomitant statin use. Both cases showed symptom resolution within 72 hours of discontinuation of medication, and labs were normal at the 2-week follow-up [13,14]. 

Across phase 2 and 3 trials with once-daily dosing of 4 mg/kg/kg/day or 6 mg/kg/day, only two out of 1342 patients (0.2%) developed daptomycin-related muscle side effects, including significantly elevated CK levels or myalgia and muscle weakness. All patients improved upon discontinuation of the medication [13].

In a retrospective study of 79 patients with daptomycin treatment, six had increased CK levels >600 U/L, and five out of the six patients had underlying renal failure, obesity, or doses >6 mg/kg/day. Two of these patients had statin use. CKD, obesity, sepsis, and concurrent statin use are identified as risk factors [15]. In another retrospective study of 126 patients dosed according to their actual body weight, patients with obesity had a CK level >1000 in 8.4% of cases. However, only one patient had clinically significant rhabdomyolysis nine days after use, but the patient was notably on simvastatin [16]. King ST et al. reported a patient with a BMI of 36 kg/m2, presenting with renal failure, liver injury, and rhabdomyolysis after daptomycin use [8]. Data on the exact role of obesity in this association is limited.

Most patients develop rhabdomyolysis within a few days, but the recovery typically occurs within a few days to a couple of weeks of discontinuing the medication. It is recommended to avoid nephrotoxins and treat with intravenous fluids as feasible. Previous case reports showed that CK levels and liver enzymes might increase even higher for a few days after discontinuing the medication. However, most cases have clinical and laboratory improvement subsequently.

Caution is advised in patients with underlying chronic kidney disease (CKD), obesity, and hypoalbuminemia. We also suggest caution in overweight patients (BMI 25-29.9) and fluid overload states that can increase fluid body weight, since the medication is dosed based on actual body weight. Statins should be avoided when possible, as the risk of myopathy is higher in patients with concurrent statin use. These patient populations may benefit from closer monitoring and discontinuation of medication at the earliest sign of myopathy. Previous case reports have shown that alternate agents, such as vancomycin, linezolid, tigecycline, doxycycline, etc., have been safely used if daptomycin-related complications occurred.

Rifampin, by itself, is sometimes (1-2%) associated with a risk of hepatotoxicity [17]. However, in a prospective study design of 16 patients assessing the safety of the combination of daptomycin at 8 mg/kg/day (higher dose than usual) and rifampicin for osteoarticular infections, none developed safety concerns with rhabdomyolysis, acute hepatic injury, or renal failure [18].

In our case report, the patient had CKD, hypoalbuminemia, and a fluid overload state with a BMI of 28 Kg/m2. He was treated with 6 mg/kg/q24 dosing. About 11 days after starting daptomycin and rifampin, he was identified to have rhabdomyolysis and had a concurrent drug-induced liver injury and acute renal failure despite avoiding statin. It is possible that concurrently used rifampin contributed to liver injury. We used cautious IV fluid administration (50 cc/hour) due to the patient's comorbidities, including hyponatremia and congestive heart failure (CHF). Renal failure, CK levels, and liver enzymes improved a few days after discontinuing the medication and supportive treatment despite his other serious comorbidities. This shows that patients have a good prognosis if diagnosed and treated early.

## Conclusions

Daptomycin is a commonly used antibiotic, and it can be used synergistically with rifampin for the treatment of MRSA infections. Further large-scale studies are needed to evaluate the safety of this combination and identify the risk factors that can increase the risk of adverse events. Frequent monitoring is necessary to ensure patient safety in specific patient populations. Prescribing physicians should consider the risks and benefits of daptomycin and rifampin in these populations. Early diagnosis and identification of relevant symptoms and changes in CK levels are crucial in preventing serious adverse effects, but fortunately, the prognosis of adverse events appears to be good.

## References

[1] Liu C, Bayer A, Cosgrove SE (2011). Clinical practice guidelines by the infectious diseases society of america for the treatment of methicillin-resistant Staphylococcus aureus infections in adults and children: executive summary. Clin Infect Dis.

[2] Zimmerli W, Sendi P (2019). Role of rifampin against staphylococcal biofilm infections in vitro, in animal models, and in orthopedic-device-related infections. Antimicrob Agents Chemother.

[3] Thabit AK, Fatani DF, Bamakhrama MS, Barnawi OA, Basudan LO, Alhejaili SF (2019). Antibiotic penetration into bone and joints: an updated review. Int J Infect Dis.

[4] Cirioni O, Mocchegiani F, Ghiselli R (2010). Daptomycin and rifampin alone and in combination prevent vascular graft biofilm formation and emergence of antibiotic resistance in a subcutaneous rat pouch model of staphylococcal infection. Eur J Vasc Endovasc Surg.

[5] Kelesidis T, Humphries R, Ward K, Lewinski MA, Yang OO (2011). Combination therapy with daptomycin, linezolid, and rifampin as treatment option for MRSA meningitis and bacteremia. Diagn Microbiol Infect Dis.

[6] Heidary M, Khosravi AD, Khoshnood S, Nasiri MJ, Soleimani S, Goudarzi M (2018). Daptomycin. J Antimicrob Chemother.

[7] Nishimura Y (2022). Daptomycin-related rhabdomyolysis complicated by severe hyperkalemia and acute kidney injury. Cureus.

[8] King ST, Walker ED, Cannon CG, Finley RW (2014). Daptomycin-induced rhabdomyolysis and acute liver injury. Scand J Infect Dis.

[9] Kazory A, Dibadj K, Weiner ID (2006). Rhabdomyolysis and acute renal failure in a patient treated with daptomycin. J Antimicrob Chemother.

[10] Patel SJ, Samo TC, Suki WN (2007). Early-onset rhabdomyolysis related to daptomycin use. Int J Antimicrob Agents.

[11] Abraham G, Finkelberg D, Spooner LM (2008). Daptomycin-induced acute renal and hepatic toxicity without rhabdomyolysis. Ann Pharmacother.

[12] Tally FP, Zeckel M, Wasilewski MM, Carini C, Berman CL, Drusano GL, Oleson FB Jr (1999). Daptomycin: a novel agent for Gram-positive infections. Expert Opin Investig Drugs.

[13] Arbeit RD, Maki D, Tally FP, Campanaro E, Eisenstein BI (2004). The safety and efficacy of daptomycin for the treatment of complicated skin and skin-structure infections. Clin Infect Dis.

[14] Sauermann R, Rothenburger M, Graninger W, Joukhadar C (2008). Daptomycin: a review 4 years after first approval. Pharmacology.

[15] Kishimoto E, Ota L, Solomon C (2013). Daptomycin and incidence of elevated creatinine phosphokinase (CPK) levels: a case report and case series review. Hawaii J Med Public Health.

[16] Bookstaver PB, Bland CM, Qureshi ZP, Faulkner-Fennell CM, Sheldon MA, Caulder CR, Hartis C (2013). Safety and effectiveness of daptomycin across a hospitalized obese population: results of a multicenter investigation in the southeastern United States. Pharmacotherapy.

[17] Tostmann A, Boeree MJ, Aarnoutse RE, de Lange WC, van der Ven AJ, Dekhuijzen R (2008). Antituberculosis drug-induced hepatotoxicity: concise up-to-date review. J Gastroenterol Hepatol.

[18] Jugun K, Vaudaux P, Garbino J, Pagani L, Hoffmeyer P, Lew D, Uçkay I (2013). The safety and efficacy of high-dose daptomycin combined with rifampicin for the treatment of Gram-positive osteoarticular infections. Int Orthop.

